# A Deep Learning Approach for Chromium Detection and Characterization from Soil Hyperspectral Data

**DOI:** 10.3390/toxics12050357

**Published:** 2024-05-11

**Authors:** Chundi Ma, Xinhang Xu, Min Zhou, Tao Hu, Chongchong Qi

**Affiliations:** 1 School of Resources and Safety Engineering, Central South University, Changsha 410083, China; machundi456@csu.edu.cn (C.M.); x578817460@163.com (X.X.); 225512080@csu.edu.cn (M.Z.); 225512073@csu.edu.cn (T.H.); 2 School of Metallurgy and Environment, Central South University, Changsha 410083, China; 3 Fankou Lean-Zinc Mine, NONFEMET, Shaoguan 511100, China

**Keywords:** soil hyperspectral, deep learning, chromium, sensitive bands

## Abstract

High levels of chromium (Cr) in soil pose a significant threat to both humans and the environment. Laboratory-based chemical analysis methods for Cr are time consuming and expensive; thus, there is an urgent need for a more efficient method for detecting Cr in soil. In this study, a deep neural network (DNN) approach was applied to the Land Use and Cover Area frame Survey (LUCAS) dataset to develop a hyperspectral soil Cr content prediction model with good generalizability and accuracy. The optimal DNN model was constructed by optimizing the spectral preprocessing methods and DNN hyperparameters, which achieved good predictive performance for Cr detection, with a correlation coefficient value of 0.79 on the testing set. Four important hyperspectral bands with strong Cr sensitivity (400–439, 1364–1422, 1862–1934, and 2158–2499 nm) were identified by permutation importance and local interpretable model-agnostic explanations. Soil iron oxide and clay mineral content were found to be important factors influencing soil Cr content. The findings of this study provide a feasible method for rapidly determining soil Cr content from hyperspectral data, which can be further refined and applied to large-scale Cr detection in the future.

## 1. Introduction

Chromium (Cr) contamination is a major global environmental problem [1]. With increasing urbanization, there has been a pronounced rise in industrial waste discharge from mining, metallurgy, and electronic equipment manufacturing industries, which has resulted in the continuous accumulation of Cr in the natural environment. For example, indiscriminate waste discharge from tanneries in India’s Vellore district resulted in more than 65% of tested soil samples exceeding the maximum permissible Cr content limits established by environmental protection agencies [2,3,4,5]. Given the non-degradable and highly toxic nature of Cr, it poses a significant threat to the ecological environment [6,7].

Cr occurs stably in nature [8]. Cr can accumulate in living organisms by contaminating soil and water resources and then biomagnifying through the food chain [9,10]. In plants, high Cr content can damage cells and inhibit growth [11], while in humans, Cr can cause skin system, liver, and kidney dysfunction and even cancer [12,13]. Different countries have set different soil Cr thresholds (e.g., 100 mg/kg for Romania and 90 mg/kg for China), which shows that soil Cr content is a key environmental risk factor [14,15]. Therefore, detecting Cr content in soil is essential for evaluating soil Cr contamination and subsequent soil remediation.

Although it is one of the most widely used techniques for soil Cr detection, laboratory chemical analysis has some limitations, including slow speed, high cost, and environmental contamination by chemical reagents [16]. These issues would be worsened in the case of large-scale Cr studies, which are needed given the global scale of Cr pollution issues [17]. As an alternative to laboratory-based analytical methods, reflectance spectroscopy is increasingly being used to measure soil quality [18]. This method primarily relies on the principle that energy is absorbed or reflected by the vibrations of molecular bonds. This technique leverages the numerous advantages of spectroscopic measurements, including their rapid, non-destructive, cost-effective, and environmentally friendly nature, especially when processing numerous samples [19]. At present, most physical and chemical properties of soils can be predicted from soil spectra; however, there is still a significant knowledge gap in the detection of soil Cr content from soil spectra [19,20].

Soil spectra can be considered non-specific and high-dimensional data due to the overlapping reflectance signatures of many soil components [21,22,23,24]. In contrast to the commonly used principal component regression and partial least squares regression techniques, deep learning-based methods, which are directly trained on the data to predict the desired soil properties, are attracting increasing attention [25,26,27,28]. The nonlinear nature of deep learning makes it well suited to handling large volumes of high-dimensional data, and this approach has been successfully applied to similar environmental fields [29,30]. However, few studies to date have applied deep learning to Cr detection from soil spectra.

To address this research gap, this study employed a deep neural network (DNN) method to predict soil Cr content from soil spectra. As an important algorithm in the field of deep learning, the DNN approach can effectively handle large volumes of data with high dimensionality [31]; thus, this approach is suitable for large-scale Cr detection. The Land Use and Cover Area frame Survey (LUCAS) dataset was used to train the DNN model, whose performance was verified by several evaluation metrics. The trained DNN model was then interpreted to identify the spectral bands sensitive to Cr content.

## 2. Materials and Methods

As noted above, the LUCAS 2009 dataset and corresponding Cr content values were used to train and validate the DNN model. To determine the optimal spectral preprocessing methods for Cr detection, the first derivative, second derivative, convolution smoothing, and multivariate scattering correction approaches were compared. The optimal model’s network structure and hyperparameters were determined using a grid search method. Model interpretation on the optimal DNN model was then performed to identify the spectral bands that were most sensitive to Cr content in soil.

### 2.1. Dataset

#### 2.1.1. Data Collection

**Figure 1:**
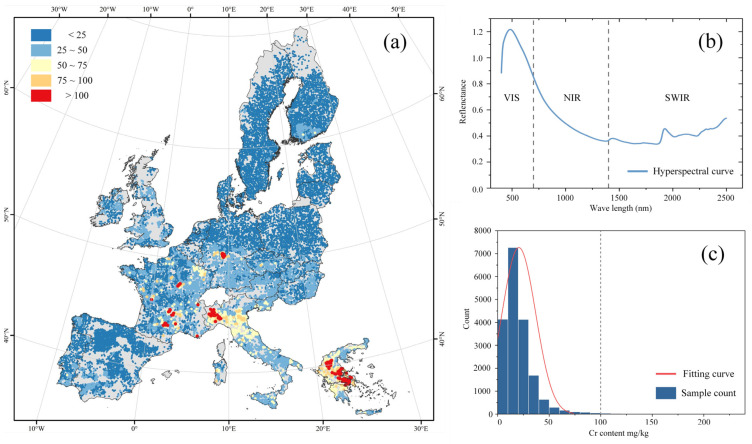
Descriptive diagram of the dataset: (**a**) Geological map of the sampling points; (**b**) Schematic diagram of the hyperspectral curve; (**c**) Distribution of Cr content.

This study used the LUCAS 2009 dataset from the European Soil Data Center (ESDAC) with topsoil samples from 23 EU member states. A total of 18,675 topsoil samples were employed based on the LUCAS project (Figure 1a). In the original acquisition of this dataset, the samples from these points were collected using a standardized sampling procedure and sent to the same laboratory for physical and chemical analysis [32,33]. The topsoil samples were ground, dried, and scanned using an XDS™ Rapid Content Analyzer (Foss, Hillerød, Denmark) to obtain reflectance spectra data in the wavelength range of 400–2500 nm with a resolution of 0.5 nm; thus, the reflectance of 4200 wavelengths were measured in this region [34]. The hyperspectral curve is schematically illustrated in Figure 1b.

The LUCAS 2009 dataset has immense value for understanding soil characteristics and properties across the EU member states. By incorporating a large quantity of topsoil samples systematically collected from diverse locations, this dataset provides a comprehensive overview of soil variations and compositions within the EU. The standardized sampling procedure ensures data consistency and reliability across a large geographic area, making this dataset a valuable resource for soil-related studies.

The application of hyperspectral analysis enables detailed characterization of the reflectance spectra of the topsoil samples. This analysis allows for the identification of specific wavelengths that correspond to various soil properties and components. The reflectance spectra data, which span a broad range of wavelengths, provide important insights into the chemical, physical, and mineralogical properties of the topsoil samples [35]. Overall, the LUCAS 2009 dataset can be used to gain a deeper understanding of soil variability, nutrient content, organic matter composition, and other essential factors that influence soil health and fertility [36]. The rich and spatially extensive data provided by the LUCAS 2009 dataset can be used for in-depth soil studies, statistical modeling, and the development of predictive models to enhance soil management practices, land use planning, and environmental assessments.

The soil Cr content values were extracted from Cr maps [37]. Note that the Cr measurements were collected at the same sampling sites as the LUCAS 2009 study, ensuring consistency when integrating these two datasets. The distribution of Cr content indicates that most of the samples were not contaminated, when a Cr contamination threshold of 100 mg/kg was used (Figure 1c) [38]. The contaminated samples were primarily concentrated in countries located in South and South Central Europe, such as Italy and Greece.

#### 2.1.2. Spectral Preprocessing Methods

**Figure 2:**
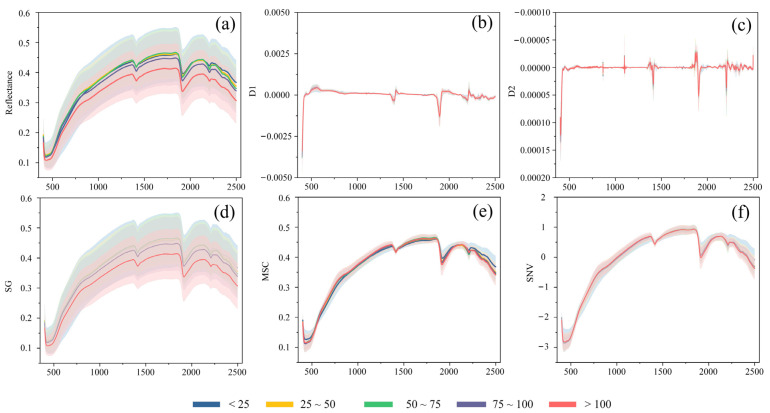
Hyperspectral curves with different spectral preprocessing: (**a**) Original spectral curve; (**b**) D1 preprocessed curve; (**c**) D2 preprocessed curve; (**d**) SG preprocessed curve; (**e**) MSC preprocessed curve; (**f**) SNV preprocessed curve. The curve represents the average value for each corresponding Cr group and the shadow represents the standard deviation.

Spectral preprocessing techniques can transform reflectance measurements using various mathematical methods that remove physical variability from light scattering and enhance features of interest [39]. Many studies have shown that applying appropriate spectral preprocessing can improve model accuracy [40,41,42]. In this study, five commonly used spectral preprocessing methods were applied and compared, including the first-order derivative (D1), second-order derivative (D2), Sazitzky–Golay (SG), multiplicative scattering correction (MSC), and standard normal variate normalization (SNV) (Figure 2) [43].

All five hyperspectral preprocessing techniques can potentially enhance hyperspectral curve quality and analytical precision [44,45,46,47,48]. D1 and D2 effectively counteract baseline drift, refining peak recognition and quantification accuracy [49]. The advantage of SG smoothing is its ability to eliminate noise without altering peak attributes and improve signal-to-noise ratios and resolution; however, excessive smoothing can potentially worsen the peak definition [50]. The MSC method tackles scattering variations, amplifies spectrum-content correlations, and refines quantitative analysis, but its benefits may be limited in non-scattering-dominant scenarios [4]. SNV promotes spectrum comparability and aids feature identification; however, this technique may cause noise amplification, particularly in low-intensity zones, which can in turn influence subsequent interpretations [51]. These preprocessing methods can be applied to optimize specific data attributes and objectives and improve hyperspectral analysis precision; however, a comparison of these techniques is essential for optimal Cr detection.

### 2.2. Deep Learning

#### 2.2.1. DNN Architecture

**Figure 3:**
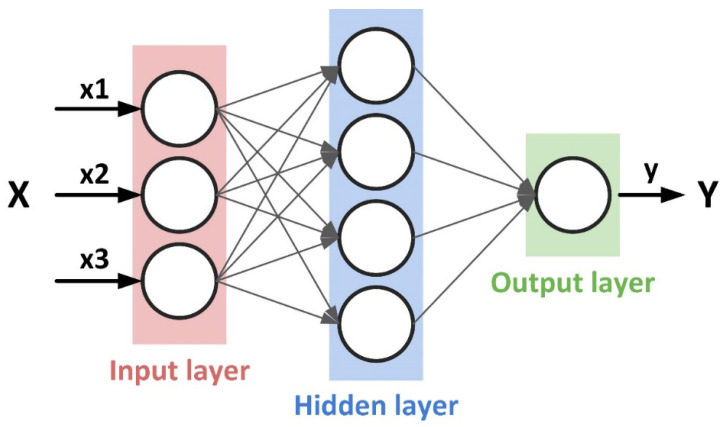
Schematic illustration of the DNN.

DNN models consist of multiple processing layers used to learn from and compute data [52]. Compared to other common regression models, the DNN approach is uniquely suited to compute large data with high dimensionality [53]. A DNN is composed of three main parts: an input layer, hidden layers, and an output layer, where each neuron within a layer is interconnected with all the neurons from the preceding layer, and nonlinearity is introduced by using activation functions on these connections (Figure 3) [54]. Each layer contains a specified number of neurons, and each neuron receives a set of inputs, which are weighted and aggregated then transformed via the activation function.

Activation functions are key components within a DNN, introducing non-linearity and enabling the network to capture complex patterns [55]. The selection of activation functions depends on the data characteristics and network architecture. Selecting suitable activation functions can significantly enhance the DNN’s ability to capture intricate relationships in hyperspectral data, which can contribute to accurate Cr prediction [56].

Layers represent the fundamental building blocks of a DNN and facilitate the extraction of hierarchical and abstract features from raw data. Each layer in a DNN serves as a specialized processing unit, transforming the input data through learned weights and activation functions [57]. The overall DNN structure typically consists of an input layer, multiple hidden layers, and an output layer. The data are received through the input layer, and feedforward processing is performed, during which the difference between the predicted and true values is calculated using a loss function. The parameter weights of each layer are then updated by an optimizer using backpropagation to minimize the misfit between the model predictions and true values. [58].

#### 2.2.2. DNN Structure and Parameter Optimization

The construction of ML models must be optimized to achieve ideal performance on specific problems [59]. Both the DNN’s structure and its hyperparameters will directly affect modeling performance. When a DNN is trained, the data are usually computed in batches, and the batch size will affect the model’s training efficiency and generalization ability. Batch size is the number of training samples used in one iteration and plays a critical role in the training efficiency of a DNN. A larger batch size can accelerate the training process as more data are processed simultaneously, leading to faster convergence. However, this speed comes at the cost of accuracy. Larger batches provide a less accurate estimate of the gradient, potentially leading the training process to converge to suboptimal solutions. Conversely, a smaller batch size tends to provide a more accurate gradient estimate, enhancing the model’s ability to generalize but slowing down the training process. Therefore, selecting an optimal batch size represents the balance between training speed and model accuracy. The larger the batch size, the faster the model’s training speed but the lower its accuracy.

The dropout rate aims to reduce the risk of model overfitting by randomly dropping some neurons during the training process so that each neuron is more independent. During training, a randomly selected subset of neurons is ignored or ‘dropped out’. This process prevents neurons from co-adapting too much, encouraging individual neurons to learn features independently, thereby reducing the model’s reliance on any small set of neurons and thus mitigating overfitting. However, setting the dropout rate too high can lead to underfitting, where the model fails to learn the data’s underlying pattern adequately. Thus, the dropout rate must be carefully calibrated to ensure the model learns sufficiently complex patterns without overfitting to the training data. Therefore, it is necessary to carefully optimize the batch size and dropout rate hyperparameters to enhance the model’s performance.

In this study, the number of layers, the number of neurons, the activation function, batch size, dropout, and learning rate were optimized using the grid search approach (Table 1) [60]. The performance of each combination was calculated as the average of 10 repetitions to avoid the effect of randomness during dataset splitting. The dataset was split in an 8:1:1 ratio of training, validation, and testing sets [61].

#### 2.2.3. Model Evaluation Metrics

Model evaluation is an important step in ML modeling and different evaluation metrics focus on different aspects of the trained models. In this study, the Pearson coefficient (R), root mean square error (RMSE), and mean absolute error (MAE) were chosen to evaluate the model’s performance [62]. R measures the correlation between the model’s predicted variables and the actual variables, thereby assessing the quality of the model’s predictions. The RMSE is the square difference between the true and predicted values and represents the magnitude of the error generated in the model’s predictions. The MAE is the average absolute difference between the true and predicted values. In general, lower RMSE and MAE values and higher R values indicate better model performance. The above metrics are calculated as follows:

(1) $$R=\frac{Cov(X,Y)}{\sqrt{Var\left[X\right]Var[Y]}}$$

(2) $$RMSE=\sqrt{\frac{1}{n}\sum_{i=1}^{n}{\left(y_{i}-{\hat{y}}_{i}\right)}^{2}}$$

(3) $$MAE=\sum_{i=1}^{n}\frac{\left|x_{i}-y_{i}\right|}{n}$$

where n is the number of samples, $y_{i}$ is the true Cr content of soil sample i, and ${\hat{y}}_{i}$ is the Cr content predicted by the model for sample i.

### 2.3. Model Interpretation

#### 2.3.1. Overview of Model Interpretation

Deep learning models have proven to be highly effective in processing large-scale datasets, but their inherent “black box” nature often obscures the understanding of the internal mechanics influencing their results. This opacity can cast doubts on their reliability, especially in critical applications. To address this, the development of interpretable methods has become essential, providing insights into how these models arrive at their conclusions [63].

Interpretable methods in machine learning are broadly categorized into two types: global and local interpretable methods. Global interpretable methods aim to identify the overall internal working mechanisms of deep learning models. These methods are designed to provide a comprehensive view of how input data are transformed and processed through various layers and nodes of a neural network. By understanding these global mechanisms, it becomes possible to gain insights into the model’s overall decision-making process, enhancing the transparency and trustworthiness of the model. In contrast, local interpretable methods focus on elucidating the causal relationships between specific inputs and their corresponding model predictions. They break down the prediction process for individual instances, enabling researchers to understand why a model made a particular decision for a specific input. This level of granular insight is invaluable for diagnosing and refining models, especially when dealing with complex datasets where the interactions between input variables can be intricate and non-intuitive [64]. In summary, interpretable models enhance the transparency and credibility of deep learning models, offering avenues for processing and optimizing the models.

#### 2.3.2. Permutation Importance

Permutation importance is a valuable and insightful feature importance assessment technique that plays a crucial role in understanding and interpreting the outputs of machine learning (ML) models. This method, based on the predicted outputs of the ML model, offers a straightforward yet powerful way to determine the significance of different features in the model’s predictions [65]. The process of permutation importance involves a systematic alteration of each feature in the dataset. To assess the importance of a particular feature, that feature’s values are shuffled or ‘permuted’, while keeping the values of all other features unchanged. This shuffling disrupts the relationship between the feature and the target, essentially simulating a scenario where the feature does not provide any useful information to the model.

Once the data with the disrupted feature is prepared, it is fed back into the model for prediction. The key step in permutation importance is comparing the model’s predictions on this perturbed data against its predictions on the original, unaltered data. The difference in performance, typically measured in terms of accuracy or error, indicates how much the model relies on the feature. A large degradation in the model’s performance upon permuting a feature indicates its high importance. Conversely, if the model’s performance remains relatively unchanged, the feature is likely less important or even redundant. This method of evaluating feature importance has several advantages. Firstly, it is model-agnostic, meaning it can be applied to any ML model regardless of its internal mechanics. This makes permutation importance particularly versatile and widely applicable across various types of models and algorithms. Secondly, it is computationally efficient, often requiring only a few additional rounds of prediction, making it suitable for large datasets and complex models [66,67]. Furthermore, permutation importance provides a more intuitive understanding of feature importance compared to other methods like coefficients in linear models or feature importance scores in tree-based models. It helps in identifying not just the highly influential features but also those which might be misleading or non-informative. This can guide the feature selection process, leading to simpler, more interpretable, and often more generalizable models.

#### 2.3.3. Local Interpretable Model-Agnostic Explanations (LIME)

The main concept of the LIME method is to interpret a complex model by constructing a simple model. This method begins by perturbing the input data and creating a new, representative dataset that reflects the original data distribution and characteristics. The newly generated dataset is then used to train a simpler model, which is inherently more interpretable than the complex model. The key objective of LIME is to ensure that the predictions made by this simpler model on the new dataset closely align with those made by the complex model on the same data. By achieving this alignment, LIME effectively reveals how the complex model behaves locally around specific instances. It also verifies the local fidelity of the simple model to the complex global model. The goal of this method is to identify the features with a high degree of importance to the model [68]. The underlying principle of LIME is shown in Equation (4). To make the local model more accurately represent the global complex model, the error between the predicted values of the global complex model *f* and the new simple model *g* must be minimized. Subsequently, the locality-aware loss function is constructed as Equation (5).

(4) $$Explanation\left(x\right)=\arg\underset{g\in G}{\min}L\left(f,g,\pi_{x}\right)+\Omega \left(g\right)$$

(5) $$L\left(f,g,\pi_{z}\right)=\sum_{z,z^{\prime }\in Z}\pi_{x}(z)(f\left(z\right)-g(z^{\prime }){)}^{2}$$

where f denotes the global complex model, i.e., the model to be explained, g denotes the simple model, G is a collection of simple models, e.g., all possible linear models, $\pi_{x}$ denotes the proximity measure of data $z^{\prime }$ in the new dataset to the original data z, and Ω(g) denotes the complexity of simple model g.

### 2.4. Implementation and Visualization

Python 3.8 was used as the programming language in the current study. Spectral preprocessing calculations were conducted using NumPy 1.25.2, Pandas, and SciPy 1.11.4. Keras 2.15.0 and scikit-learn 1.2.2 were utilized for the establishment, training, and performance assessment of the DNN models. The interpretability analysis of the models was conducted using Lime 0.2.0.1, SHAP 0.42.0, and eli5 0.13.0.

## 3. Result and Discussion

### 3.1. Model Optimization Results

**Figure 4:**
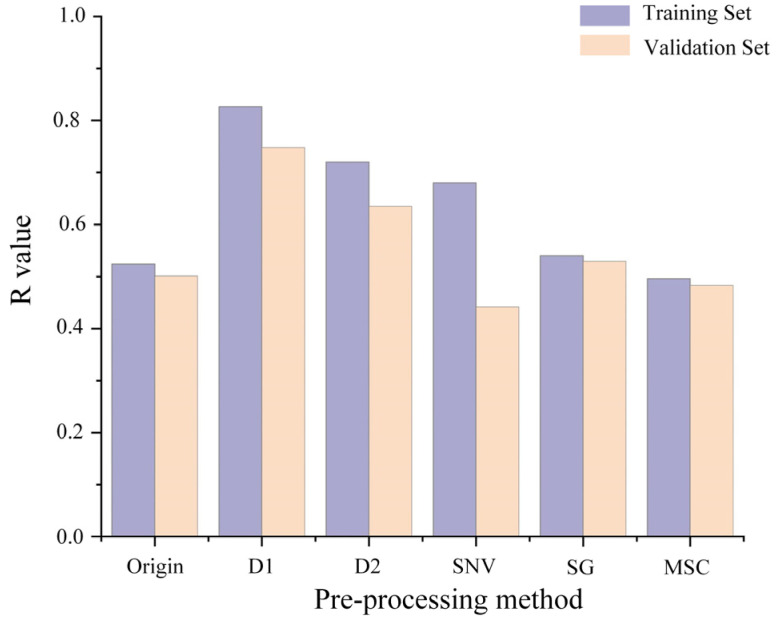
The influence of spectral preprocessing on DNN modeling performance.

In this study, the influence of five preprocessing methods (SNV, SG, D1, D2, SG, and MSC) on the modeling performance was compared based on the R value, as shown in Figure 4. D1 performed optimally on both the training and validation sets, with R values of 0.83 and 0.75, respectively. Compared to the original spectra, the DNN model trained on D1 preprocessed spectra exhibited a significant improvement, achieving the R increase of 0.30 in the training set and 0.247 in the testing set.

**Figure 5:**
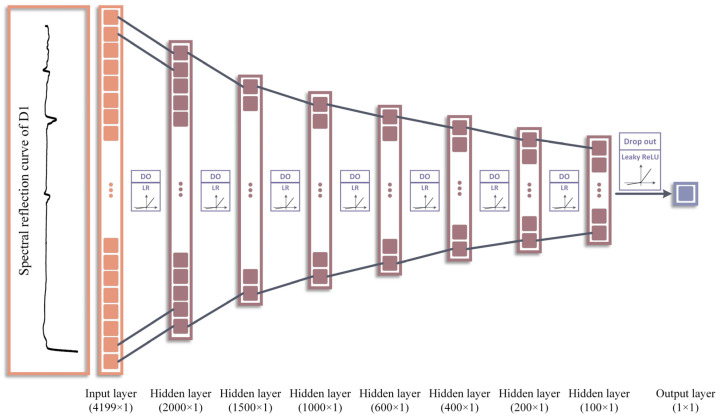
The optimized DNN structure.

To obtain the optimal DNN structure, the number of hidden layers and the number of neurons in the hidden layer were adjusted [59]. After the architecture optimization, the selected DNN structure comprised seven hidden layers. These layers contained 2000, 1500, 1000, 600, 400, 200, and 100 neurons, respectively, and were connected by the Leaky ReLU activation function. To prevent gradient explosion and overfitting, the dropout and early stopping mechanisms were also used during DNN model construction (Figure 5) [69,70].

**Figure 6:**
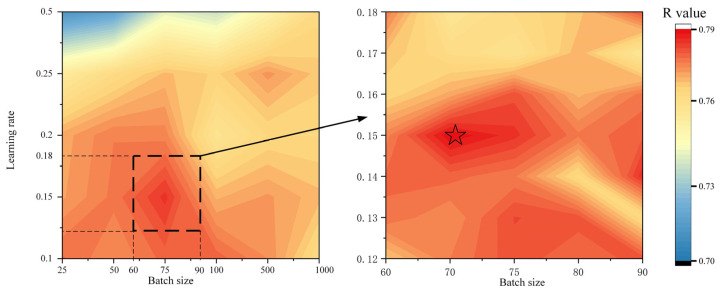
The optimized batch size and learning rate.

As described above, different combinations of dropout rate, batch size, and learning rate will affect the modeling performance. After comparing the performance of learning rates of 0.01, 0.001, and 0.0001, a value of 0.001 was found to achieve better performance and was thus used for the subsequent optimization (Figure 6) [71]. The grid search of the dropout rate and batch size hyperparameters indicates that the model’s performance was optimized when the dropout rate was 0.15 and the batch size value was 70. For the above optimal parameter combination, the DNN model achieved the R values of 0.85 and 0.79 on the training and validation sets, respectively.

### 3.2. Model Evaluation

**Figure 7:**
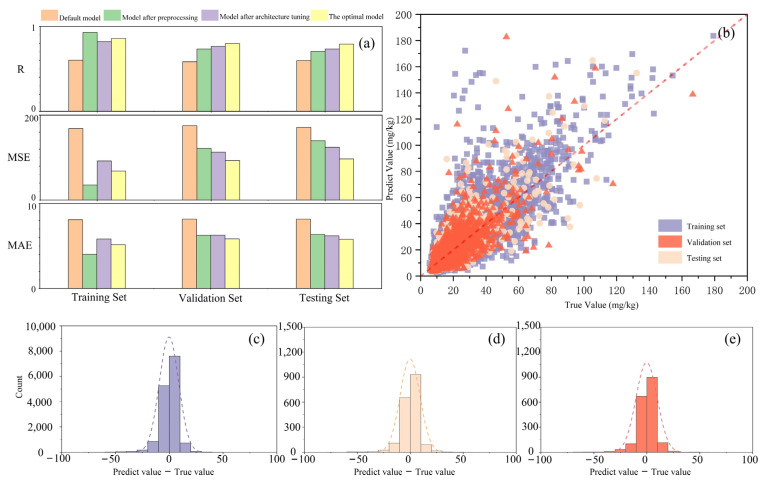
Evaluation of modeling performance: (**a**) Values of model evaluation metrics at each modeling stage; (**b**) Comparison of the actual and predicted Cr values using the optimal model; (**c**–**e**) Distribution of the difference between predicted and actual Cr values in the training, validation, and testing sets of the optimal model, respectively. The ‘default model’ refers to the initial DNN model, ‘preprocess model’ to the model post preprocessing, ‘structure model’ to the model after optimizing the neural network structure, and ‘optimal model’ to the model achieving the best performance.

Figure 7a presents a comparative analysis of the DNN model’s performance throughout the optimization process, ranging from the default model to the optimal model. A marked enhancement in the DNN’s performance was observed during the whole model construction process. Specifically, the R of the model improved from 0.6 to 0.8 on the validation set, indicating a significant increase in the correlation between the model’s predictions and the actual values (Figure 7b). In the meantime, RMSE was decreased from 168.6 to 68.4 and MSE was decreased from 8.16 to 5.84. These improvements in evaluation metrics demonstrate enhanced accuracy and reliability of the model in predicting chromium content in soil. The discrepancies between the actual and predicted values in the training, validation, and testing sets were predominantly concentrated within a range of 10, further underscoring the effectiveness of the DNN modeling (Figure 7c–e). On the testing set, the optimal DNN model exhibited robust predictive performance with an R value of 0.79, RMSE of 96.98, and MAE of 5.79. These results indicate that the model’s capability to predict chromium content from soil hyperspectral data was enhanced after the optimization of DNN architecture and hyperparameters.

### 3.3. Spatial Autocorrelation and Residual Analysis of the DNN Prediction

Considering that the soil chromium content might have some spatial patterns, Moran’s index was calculated at 0.287 with a *p*-value of 0.001 using the residuals and geographical coordinates from the DNN model. This indicates a tendency for chromium to cluster within the spatial scope of the European Union, confirming the presence of significant spatial autocorrelation in the data.

**Figure 8:**
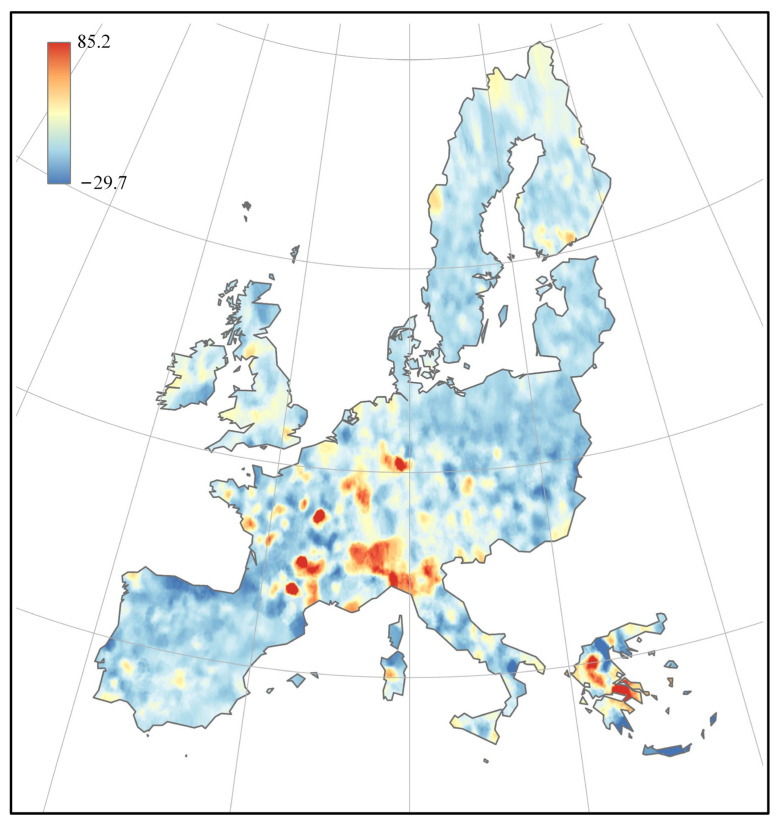
The distribution of the prediction residual across the EU.

Further analysis was performed on the residuals through kriging interpolation, as shown in Figure 8. This revealed that large residuals were predominantly distributed among Southern and Central European countries, while predictions for countries in Northern Europe were more reliable. In future research, more tailored DNN models could be established for different regions or countries within the European Union to enhance the robustness of the model’s predictions.

### 3.4. Model Interpretation Analysis

**Figure 9:**
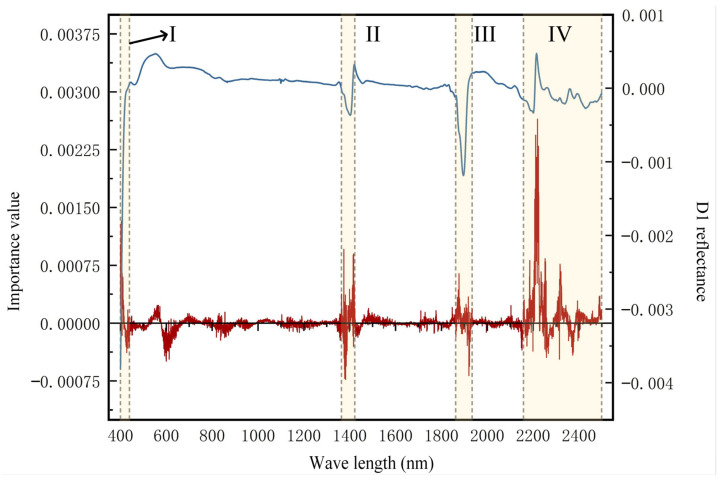
Permutation importance across the whole spectra. The blue curve is a representative hyperspectral curve after D1 preprocessing, the red curve represents the permutation importance value across the spectra, and the four regions are the sensitive band ranges.

As detailed above, the permutation importance can be calculated by randomly disrupting a single feature in all the samples. This can then be used to calculate the impact on the model’s accuracy due to the change in that feature and, thus, the importance of each feature [72]. The calculated feature permutation importance using the optimized DNN model exhibited a relatively smooth trend (Figure 9); however, there were significant fluctuations in four specific band ranges: 400–439 nm (region I), 1364–1422 nm (region II), 1862–1934 nm (region III), and 2158–2499 nm (region IV). More pronounced absorption peaks were also observed in these ranges in the hyperspectral reflection curves processed using the first-order differential method.

Among these four band regions, region I is mainly attributed to the iron oxide content, while the peaks in regions II and III are primarily related to the presence of clay minerals and hydroxyl groups in water [73]. Region IV involves vibrations of metal–OH bonds and indicates a key absorption peak near 2200 nm; this peak is mainly influenced by Al–OH bonds, with the main contributing substances being kaolinite, montmorillonite, and illite [74].

The above permutation importance results indicate that soil Cr content is mainly correlated with clay minerals and iron oxides. Clay minerals directly influence soil texture and play a crucial role in the growth of plants and microorganisms. These factors, in turn, exert a significant influence on Cr content and flow within ecosystems [75]. For example, montmorillonite, a clay mineral characterized by a layered structure and active surface sites, can adsorb Cr through processes such as ion exchange with water within its lattice or complexation on its surface [75,76]. As an iron-loving element, Cr can be adsorbed with iron oxides to produce stable precipitates or surface complexes—these promote the binding of Cr to soil particles and thus reduce damage to the environment [77,78]. Therefore, the summarized high correlation between Cr content with clay minerals and iron oxides agrees well with the findings in the literature.

**Figure 10:**
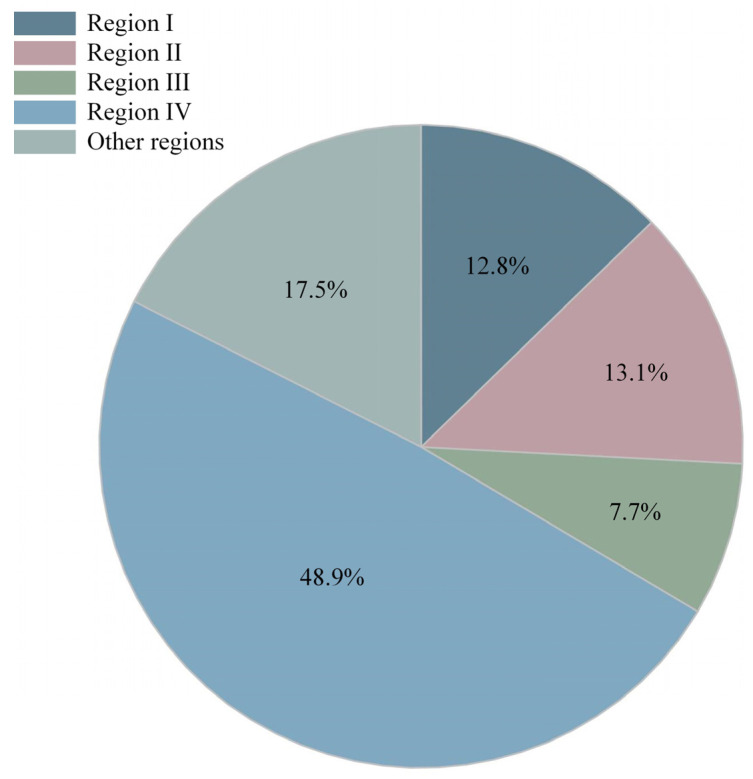
LIME importance analysis.

In this study, 1000 samples were randomly selected for LIME analysis and their 10 most important features were classified based on the four important regions identified in Figure 8. The LIME results are summarized in Figure 10. As shown, 77% of the important features identified in the LIME analysis belonged to one of the four intervals, while the other 23% were irregularly distributed in the other regions. The above results indicate a good agreement between LIME and permutation importance results. Notably, the number of important features in region IV was much higher than those in regions I, II, and III, and the top 10 most important features identified from the permutation importance were also distributed in region IV. Therefore, clay minerals were observed to be the most important indicators for Cr content in soil.

The above importance analysis indicates that the chemical properties of soil significantly affect the accumulation of Cr in soil. To prevent excess Cr accumulation in the soil, heavy metal industries should not be distributed in soils with high clay minerals and iron oxide content. This measure can help minimize the extent and impact of Cr pollution resulting from industrial accidents and improper sewage treatment. For soils that have accumulated large amounts of Cr, remediation using iron oxide or clay minerals can be performed to absorbed Cr from soil.

## 4. Conclusions

In this study, various analyses were performed based on the LUCAS dataset to establish a DNN model for Cr detection from soil spectra. As part of the optimization process, the optimal preprocessing method was determined, and the model’s hyperparameters were tuned. The resulting optimum DNN model can accurately predict soil Cr content from soil spectra. Meanwhile, four Cr-sensitive bands were identified through the interpretation using the optimal DNN model. The main conclusions are as follows:

(1) D1 was identified as the optimal preprocessing method for the DNN model to predict soil Cr content. The R value of the DNN model increased from 0.50 to 0.75 on the testing set after spectral preprocessing.
(2) The adjustment of DNN architecture and hyperparameters resulted in the further improvements in the model performance. The R, RMSE, and MAE values of the optimal model on the testing set were 0.79, 96.98, and 5.79, respectively, which were significantly improved compared to the default model.
(3) Four important sensitive band regions of Cr content in soil were identified, namely, 400–439 nm (region I), 1364–1422 nm (region II), 1862–1934 nm (region III), and 2158–2499 nm (region IV). These bands correspond primarily to iron oxide and clay mineral content in the soil.

## Figures and Tables

**Table 1:** Hyperparameter selection range.

Parameter	Search Range
Layer	[1, 2, 3, 4, 5, 6, 7, 8, 9]
Neurons	[100, 200, 400, 600, 800, 1000, 1500, 2000]
Activation function	[ReLU, Leakly_ReLU, Swish, Sigmoid]
Batch size	[25, 50, 75, 100, 500, 100]
Dropout rate	[0.1, 0.2, 0.3, 0.4, 0.5]
Learning rate	[0.01, 0.001, 0.0001]

## Data Availability

The data that has been used is confidential.
